# Campbell Title Registrations to Date–October 2020

**DOI:** 10.1002/cl2.1130

**Published:** 2020-12-29

Details of new titles for systematic reviews or evidence and gap maps that have been accepted by the Editor of a Campbell Coordinating Group are published in each issue of the journal. If you would like to receive a copy of the approved title registration form, please send an email to the Managing Editor of the relevant Coordinating Group.

## 1 CLIMATE SOLUTIONS

*Effectiveness of finance for supporting climate change adaptation in the agricultural sector in low‐ and middle‐income countries*

Biljana Macura, Richard Taylor, Nella Canales, Fedra Vanhuyse

1 October 2020

## 2 CRIME AND JUSTICE

*Mental disorder and terrorist behaviour*

Kiran M. Sarma, Sarah L. Carthy, Katie M. Cox

24 September 2020

*What works to reduce rates of homicide and robbery in Latin America and the Caribbean: A systematic review*

Alberto Liebling Kopittke, Rodrigo Serrano‐Berthet, Rodrigo Pantoja, Luciane Kopittke, Águida Luana Veriato Schultz, Fernando Gonçalves de Gonçalves

1 September 2020

*Government‐led communication‐based interventions for reducing violent extremism: A systematic review*

Kurt Braddock, Paul Gill, Sarah Carthy, Megan Swick

31 August 2020

*Hate online and in traditional media: A systematic review of the evidence for associations or impacts on individuals, audiences, and communities*

Ghayda Hassan, Jihan Rabah, Sebastien Brouillette‐Alarie, Pablo Madriaza, Eugene Borokhovski, David Pickup, Wynnpaul Varela, Melina Girard, Loïc Durocher‐Corfa, Emmanuel Danis

17 August 2020

*Police stops to reduce crime: A systematic review*

David Weisburd, Kevin Petersen, Taryn Zastrow, Robert Davis

15 August 2020

*Is radicalization a family issue? A systematic review of family‐related risk and protective factors, consequences and interventions against radicalization*

Izabela Zych, Elena Nasaescu

31 July 2020

## 3 DISABILITY

*Systematic review on evaluations of employer‐focused policies designed to improve employment opportunities and outcomes for people with disabilities*

Daniel Derbyshire, Esmaeil Khedmati Morasae, Morwenna Rogers

21 September 2020

## 4 EDUCATION

*Impact of COVID‐19 on education and human capital of children and adolescents in low‐ and middle‐income countries*

Bianca Carducci, Maryam Farooq, Anushka Ataullahjan, Emily C. Keats, Zulfiqar A. Bhutta

25 September 2020

*School interventions that strengthen school climate and school identification: A systematic review*

Diana Cárdenas, Kate J. Reynolds

25 September 2020

*Support services in provision for under‐represented groups of online students in higher education: A systematic review*

Chloe Warner (Cox), Professor Jacqueline McCormack, Dr Ellen E. A. Simpson, Dr Allison M. C. Gillen

25 September 2020

*The effect of interventions for self‐efficacy and maths self‐efficacy on the mathematics performance of adolescents and young adults in education: A systematic review*

Yasmin Bador, Charis Voutsina, Julie Hadwin

17 September 2020

## 5 INTERNATIONAL DEVELOPMENT

*Systematic review of interventions involving men in family planning in low‐ and middle‐income countries*

Áine Aventin, Martin Robinson, Jennifer Hanratty, Eimear Ruane‐McAteer, Mark Tomlinson, Mike Clarke, Friday Okonofua, Chris Bonell, Maria Lohan

1 October 2020

*Effects of women's groups on gendered asset ownership in low‐ and middle‐income countries*

Thomas de Hoop, Amber Peterman, Garima Siwach, Chinmaya Holla, Sohini Paul, Sapna Desai, Eve Namisango, Ekwaro A. Obuku

1 October 2020

*How are menstrual hygiene management (MHM) interventions providing feminine hygiene products, and what are the characteristics of such interventions to serve as sustainable solutions? A systematic review*

Sara Spencer, Alexandre Dumouza, Aryan Heydarypour

1 October 2020

*Conditional and unconditional cash transfers for health and nutritional outcomes in poor families in low‐ and middle‐income countries: A systematic review*

Lufang Feng, Xiuxia Li, Na Zhang, Nan Chen, Xiajing Chu, Jingwen Li, Jieyun Li, Kangle Guo, Peijing Yan, Xingrong Liu, Kehu Yang

1 October 2020

*The effect of digital payments for social safety nets on take‐up and transaction costs for implementers and beneficiaries in low‐ and middle‐income countries: A systematic review*

Zukarin Jahangir, Howard White, Ashrita Saran, Iffat Zahan, Maria Matin, Shamael Ahmed

1 October 2020

*WASH interventions, gender and social equality*

Biljana Macura, Laura Del Duca, Adriana Soto, Sarah Dickin

11 September 2020

## 6 METHODS

*Bibliographic database searching methods across CEE and Campbell Collaboration systematic reviews and maps: A systematic review*

Neal R. Haddaway, Ciara Keenan, Christian Kohl, Sarah Young

13 October 2020

## 7 SOCIAL WELFARE

*A systematic review of the effectiveness of mental health interventions for adult adoptees*

Anne Farina, Hollee McGinnis, Amanda Baden, Mary O'Leary Wiley, Kristen Kremer

5 October 2020

*Impact of social protection on gender equality in low and middle‐income countries: A systematic* review of reviews

Camila Perera, Shivit Bakrania, Alessandra Ipince, Zahrah Nesbitt‐Ahmed, Oluwaseun Ireti Obasola

11 September 2020

*An evidence and gap map of studies of implementation issues for interventions for those affected by and at risk of homelessness*

Ting Yang, Howard White, Ashrita Saran, Suzanne Fitzpatrick, Jenny Wood, Ligia Teixeira

18 August 2020

*Effects of virtual reality exposure therapy versus in vivo exposure in treating social anxiety disorder in adults: A systematic review and meta‐analysis*

Martin Polak, Norbert Tanzer, Per Carlbring

28 July 2020

